# Supplementary material for: Cell Dome as an Evaluation Platform for Organized HepG2 Cells
Source: Cells. 2022 Dec 23;12(1):69. doi: 10.3390/cells12010069 (PMC9818560; doi:10.3390/cells12010069)
Supplement: Supplementary file 1 [file cells-12-00069-s001.zip › cells-1972612-supplementary.pdf]

cells MDPI

Supplementary Information

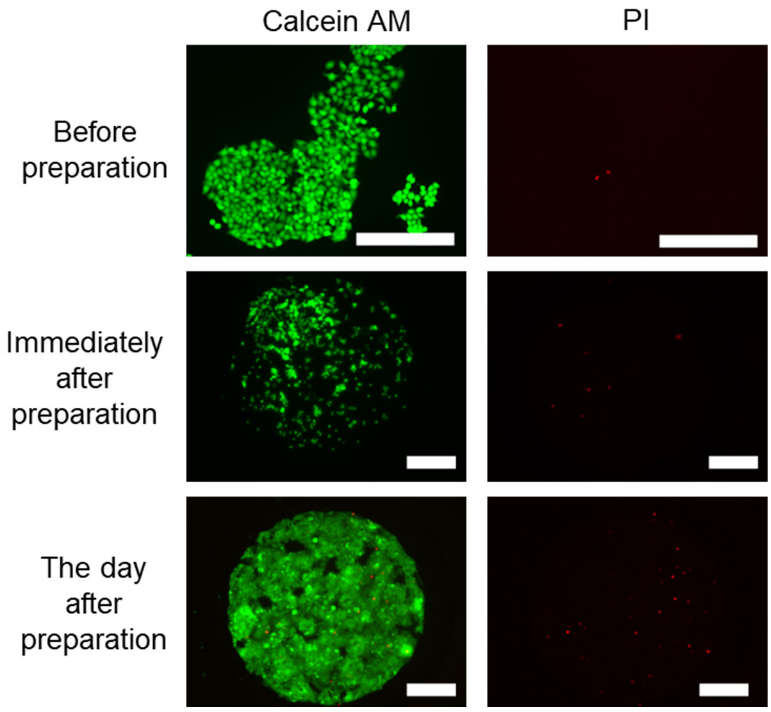

Figure S1. HepG2 cells before enclosing and, immediately and a day after enclosing stained with Calcein AM and PI indicating live and dead cells, respectively (Bars: 250 μm).
